# FSGS Recurrence in Adults after Renal Transplantation

**DOI:** 10.1155/2016/3295618

**Published:** 2016-04-10

**Authors:** Michael Rudnicki

**Affiliations:** Department of Internal Medicine IV-Nephrology and Hypertension, Medical University Innsbruck, Anichstrasse 35, 6020 Innsbruck, Austria

## Abstract

Recurrence of focal segmental glomerulosclerosis (FSGS) in the allograft occurs in 30–50% of patients, and it is associated with poor renal allograft survival. Major risk factors for recurrence are younger age at diagnosis, rapid progression to end-stage renal disease, white race, and the loss of previous allografts due to recurrence. Recent data support the hypothesis that circulating permeability factors play a crucial role in podocyte injury and progression of FSGS. Due to lack of controlled trials, the management of recurrent FSGS is inconsistent and highly empirical. Prophylactic and perioperative treatment with plasmapheresis and high-dose (intravenous) cyclosporine represent the main cornerstones of immunosuppressive therapy. In recent years, therapy with rituximab has shown promising results. Despite evidence of activation of the renin-angiotensin system (RAS) in recurrent FSGS and its association with progression, only limited data exist on the renoprotective role of RAS blockade in this setting. Further well designed studies are needed on pathogenesis risk factors and therapeutical options in FSGS and its recurrence after transplantation.

## 1. Introduction

Focal segmental glomerulosclerosis (FSGS) is the leading cause of nephrotic syndrome in the adult population. FSGS is either termed primary (i.e., idiopathic), when a specific cause cannot be identified, or secondary to a variety of etiologies, such as genetic (specific mutations of podocyte genes), viral-associated (e.g., HIV, parvovirus B19, simian virus 40, cytomegalovirus, and Epstein-Barr virus), drug-induced (e.g., pamidronate, heroin, lithium, interferon, calcineurin inhibitors, and sirolimus), and adaptive (e.g., structural-functional responses to glomerular hypertension, such as conditions with reduction of renal mass and hyperfiltration of the remaining nephrons) [1]. In general, only primary FSGS recurs following kidney transplantation.

Within 10–20 years from diagnosing a substantial proportion (approximately 40–70%) of patients with FSGS progress to end-stage renal disease (ESRD), making FSGS the most common primary glomerular disorder in the dialysis population with a prevalence of 4% [1–3]. The first case report of FSGS recurrence was published by Hoyer et al. in 1972 [4]. Currently, the reported FSGS recurrence rate averages approximately 30% [5, 6]. However, it is likely that the recurrence rates of idiopathic FSGS are even higher (up to 50%) due to the fact that the cause of ESRD is difficult to ascertain and it is often not clear if the patient had primary FSGS or FSGS related to other causes [7]. The clinical hallmark of FSGS recurrence is proteinuria, which is often diagnosed within days after transplantation, and sometimes the full picture of the nephrotic syndrome may be present [8]. Diffuse foot process effacement as detected by electron microscopy is the only initial finding of FSGS in early allograft biopsies. As shown by Chang et al. this characteristic histological feature may already appear within 1–2 hours after reperfusion, predicting the recurrence of nephrotic range proteinuria 3–7 days posttransplant with a sensitivity of 71% and a specificity of 92%. Furthermore, in this study there was an association of the degree of foot process effacement with proteinuria, suggesting a key role of podocyte injury in the pathogenesis of recurrent FSGS [9].

Among patients with biopsy-proven FSGS as cause of ESRD the recurrence of the disease is associated with an increased risk of allograft loss [10]. In a large study from the Australia and New Zealand Dialysis and Transplant Registry (ANZDATA) the incidence of allograft loss at 10 years due to recurrent FSGS was 12.7% (95% CI 7.3–21.6). Furthermore, those patients with recurrent FSGS had a twofold higher risk of allograft loss as compared to patients with other glomerulonephritides (adjusted HR 2.03, 95% CI 1.19–3.44) [11].

## 2. Pathogenesis of FSGS Recurrence

Gallon et al. reported an interesting case of FSGS recurrence after kidney transplantation [12]. A 27-year-old man with ESRD due to primary FSGS received a kidney transplant from his healthy 24-year-old sister. Despite pre- and perioperative plasmapheresis and standard immunosuppressive therapy, nephrotic range proteinuria developed on postoperative day 2. Allograft biopsy on day 6 revealed marked podocyte foot process effacement and loss of the interdigitating arrangements, consistent with recurrence of FSGS. On day 14 the renal allograft was removed due to severe hypoalbuminemia, progressive acute kidney injury, and an abdominal hematoma. After consultation of the institutional review board and obtaining informed consent, the kidney was transplanted into a 66-year-old man with ESRD caused by diabetes mellitus type 2. Within days after retransplantation kidney function improved and proteinuria decreased significantly. Furthermore, allograft biopsies performed on day 8 and 25 after retransplantation showed reversal of the glomerular lesions. This report supports the theory of a circulating factor as cause of primary FSGS, and it provides evidence that podocyte injury might be reversible at least before renal scarring occurs.

An extensive review of the pathogenesis of recurrent FSGS is beyond the scope of this chapter. In brief, the hypothesis that both primary FSGS in the native kidneys and also recurrent disease in the allograft are likely due to circulating factors or the absence of a normally present factor in the plasma is supported by several lines of evidence: first, it has been shown that pretransplant serum of patients with FSGS may increase the permeability of glomeruli to albumin* in vitro*. The serum of patients with recurrent FSGS after transplantation had significantly higher permeability values as compared to controls. Furthermore, the* in vitro* tested permeability was reduced to control values after plasmapheresis, which was associated* in vivo* with a decrease in proteinuria [13]. Second, insights from the Buffalo/Mna rat model of spontaneous FSGS further support the hypothesis of a circulating factor. The Buffalo/Mna rats develop proteinuria and present with renal histological features of human FSGS. Treatment with steroids, cyclosporine, or cyclophosphamide leads to proteinuria reduction. When a kidney from a healthy control rat is transplanted into a Buffalo/Mna rat, FSGS recurs. On the other hand, when Buffalo/Mna rat kidneys are transplanted into control rats, proteinuria and renal lesions regress [14]. Third, the frequent occurrence of a relapse of proteinuria early after transplantation, the rapid recovery of allograft function following retransplantation into a patient without FSGS, and improvement of proteinuria after plasmapheresis or rituximab suggest that injury of the podocytes is caused by a circulating factor, supposedly an autoantibody or a factor released by T cells upon interference with B cells. Finally, in genetic forms of FSGS the recurrence rate is low (but not zero). In patients with homozygous FinMajor-NPHS1 (nephrin) mutations, the recurrence of proteinuria posttransplant is probably due to preexisting antinephrin antibodies in the recipient [15]. In the case of FSGS due to homozygous NPHS2 (podocin) mutations, the existence of antipodocin antibodies has not been proven to date [16]. Jungraithmayr et al. identified genotype-phenotype correlations of NPHS2 mutations and recurrence of FSGS in a cohort of 53 children with primary FSGS. Interestingly, none of the 11 children who were homozygous or compound heterozygous for NPHS2 mutations developed a recurrence of FSGS, compared with 45% of the patients without mutations [17]. In a similar manner Weber et al. described FSGS recurrence only in one of 32 patients with two NPHS2 mutations [18]. In contrast to these findings Bertelli et al. found an equal recurrence rate in genetic and nongenetic forms of FSGS; however, they included heterozygous NPHS2 mutations in their analysis [19]. Circulating permeability factors such as the soluble urokinase receptor (suPAR) [20, 21] and autoantibodies directed against actin, adenosine triphosphate synthase, angiotensin II type 1 receptor, nephrin (NPHS1), protein tyrosine phosphatase receptor type O (PTPRO), and Thy1 have been implicated in the pathogenesis of FSGS recurrence [15, 22–24].

Although several potential permeability factors have been identified, current evidence on the nature of this factor and on the pathogenesis of recurrent FSGS remains frustratingly inconclusive and is an ongoing subject of extensive speculation.

## 3. Risk Factors for Recurrence

Several clinical risk factors have been associated with an increased risk of FSGS recurrence after transplantation (reviewed in [25]), including younger age at onset of initial disease (particularly between 6 and 15 years of age) [26, 27], rapid progression of primary FSGS to ESRD (<3 years) [28–30], white race [10], and the loss of previous allografts due to recurrence [28]. Retrospective data from the United States Renal Data System (USRDS) suggests that ethnicity and genetic background may have an impact on the risk of recurrence. In this analysis the risk of recurrent FSGS was higher in white than in nonwhite patients, and particularly white recipients of African-American kidneys had an increased risk of recurrence [10]. Patients who received pretransplant bilateral nephrectomy may experience a higher risk of recurrence [27]. It is speculated that native kidneys act as absorbers of permeability factors, although data on nephrectomy are contradictory [31].

Steroid-resistant nephrotic syndrome due to FSGS in the native kidneys may indicate lower risk of recurrence. In a recent study of 125 children with steroid-resistant nephrotic syndrome (>95% biopsy-proven FSGS), it was shown that 92.9% of those patients who initially were steroid-responsive (defined as complete remission of proteinuria on at least one episode after steroid therapy) experienced a recurrence of FSGS compared to only 30.2% of steroid-resistant patients (OR 30; 95% CI 6.62–135.86) [32].

Data are inconclusive on the role of induction therapy and initial immunosuppression and the risk of FSGS recurrence. In a retrospective single center study the use of antilymphocyte sera, particularly antithymocyte globulin (ATG), was associated with a higher risk of recurrence, as compared to no induction therapy [33]. On the contrary, Pascual et al. showed that induction therapy with ATG was associated with a reduced risk of recurrence of any primary glomerulonephritis, including FSGS, as compared to alemtuzumab or interleukin-2 receptor antagonists [34]. No differences in the rate of graft loss due to recurrent FSGS were seen in a large retrospective analysis (*n* = 4502 patients with FSGS as initial disease) from the Organ Procurement and Transplant Network/United Network of Organ Sharing (OPTN/UNOS) between those patients treated with mycophenolate mofetil versus those treated with azathioprine in cyclosporine based regimens [35].

Five morphological variants of FSGS exist, namely, NOS (not otherwise specified), perihilar, cellular, tip, and collapsing variant [36]. Although it has been recognized that the histological subtype predicts the course of disease in the native kidneys, it is not clear if the same subtype is observed when FSGS recurs in the allograft [37, 38]. One can speculate that recurrent FSGS in the allograft may initially represent the same histological variant as in the native kidney (maybe with the same risk of progression to ESRD). However, ischemia-reperfusion injury and the effect of calcineurin inhibitors on the single graft may cause a mismatch between nephron number and metabolic demand leading to a mixture of primary and adaptive (i.e., secondary) form of FSGS thus changing histology and prognosis [39].

As mentioned above the risk of recurrence is low in genetic or familial forms of FSGS, depending on the disease causing mutation [15, 17, 29].

Several molecules have been proposed as biomarkers to quantify the risk of recurrence after transplantation. One of the most extensively studied candidates is suPAR, which not only serves as a mere biomarker, but also has been proposed as being involved in the pathogenesis of FSGS by activating podocyte *β*3 integrin causing foot process effacement [21]. Higher levels of suPAR before transplantation were associated with an increased risk of recurrence of FSGS in the allograft [21]. Reduction of very high suPAR levels during FSGS recurrence by a combination of plasmapheresis and immunoadsorption was associated with remission of proteinuria [40], while immunoadsorption alone did not alter suPAR levels [41]. However, elevated suPAR levels have also been identified in a wide range of inflammatory diseases, including pneumonia, malaria, tuberculosis, HIV, sepsis, and also in various cancers [42]. Furthermore, the role of suPAR as a marker of FSGS and its recurrence was questioned by data showing no difference in suPAR levels between primary FSGS, secondary FSGS, and also minimal change disease (MCD). Also response to steroid therapy was not predicted by suPAR levels [43]. However, in this report an inverse correlation of suPAR levels with eGFR was found, independently of histological diagnosis. Recently, suPAR was identified also as a predictor of incident chronic kidney disease in the Emory Cardiovascular Biobank Cohort [44]. These inconclusive results can be explained by the fact that not all molecular forms of suPAR are equally pathogenic to podocytes or can be measured easily in human subjects [45], questioning the straightforward clinical usefulness of suPAR as a marker of FSGS recurrence.

In a recent study Delville et al. identified a circulating antibody panel as predictor of FSGS recurrence [46]. By high-throughput screening of pretransplant sera from patients with recurrent FSGS and patients without recurrence, the authors identified antibodies targeting glomerular antigens. Of these a panel of seven antibodies (CD40, PTPRO, CGB5, FAS, P2RY11, SNRPB2, and APOL2) was validated in an independent cohort of patients with and without FSGS recurrence. The panel was able to predict FSGS recurrence with 92% accuracy. Of these antibodies anti-CD40 alone had the best correlation with risk of FSGS recurrence (78% accuracy). Purified anti-CD40 antibodies from humans with recurrent FSGS disrupted the cytoskeleton of podocytes—possibly in a suPAR dependent manner—and also induced proteinuria in murine experiments, suggesting a role of CD40 perturbations also in the pathogenesis of FSGS and its recurrence.

## 4. Treatment of Recurrent FSGS

Despite advances in the understanding of FSGS recurrence and progression, management remains difficult and treatment decisions are often based on evidence from small case series. Data from controlled trials comparing the efficacy of various approaches are lacking.

One of the most commonly used therapies for recurrent FSGS is plasmapheresis. Since the original description of a successful treatment in 1985, numerous case reports and case series have been reported with varying degrees of success [47]. In established FSGS recurrence plasmapheresis can induce remission in 70% of children and in 63% of adults, as summarized by Ponticelli in a systematic review [48]. However, one can assume that the published treatment effect is overestimated due to publication bias, retrospective and uncontrolled design, and short-term follow-up. Best results seem to be achieved when plasmapheresis is started early after transplantation, immediately when recurrence becomes clinically evident. A typical plasmapheresis prescription is 1–2 times plasma volume exchanges, 3–4 treatments per week, and a total of 8–12 treatments until remission is achieved. However, in some cases weaning protocols or intensive plasmapheresis for up to several months has been reported [26]. Prophylactic plasmapheresis therapy during the perioperative period has also been proposed. Gohh et al. prospectively treated 10 patients with at least 8 plasmapheresis sessions during the perioperative period. Recipients of living donor kidneys received plasmapheresis from 1 week before to 1 week after the transplantation. Recipients of deceased donor kidneys received the first plasmapheresis 24 hours before implantation. Interestingly, FSGS recurrence was diagnosed in none (0 of 4) of the high-risk patients with rapid progression to ESRD and in only 50% (3 of 6) of the patients who lost their first graft due to recurrent FSGS. The authors concluded that these rates might be less than expected from historical reports [49]. However, other authors did not find any benefit from prophylactic treatment [26, 50]. Some case reports have suggested the combined use of plasmapheresis and immunoadsorption, but data on this treatment are very limited and difficult to interpret [51, 52].

The use of standard oral doses of cyclosporine has not been shown to prevent FSGS recurrence; however, higher intravenous doses have been associated with proteinuria reduction (reviewed in [39]). The rationale behind maintaining high cyclosporine blood levels is explained by the lipophilic characteristics of cyclosporine. Cyclosporine is incorporated into peripheral lymphocytes via binding to LDL receptors on the cell surface. High blood levels of LDL cholesterol as often seen in patients with nephrotic syndrome reduce the amount of the free drug. Thus, hypercholesterolemia inhibits the effect of cyclosporine on lymphocytes, and high blood levels may overcome this effect. In one prospective yet uncontrolled cohort study, intravenous cyclosporine at a dose of 3 mg/kg/day for 3–4 weeks, followed by an oral dose maintaining blood levels between 250 and 350 ng/mL, induced remission in 14 or 17 patients [53]. Raafat et al. have reported encouraging results with high-dose oral cyclosporine [54]. However, well-known complications of high doses of cyclosporine limit long-term safety of such a treatment.

Rituximab is a chimeric mouse/human monoclonal antibody targeting the CD20 surface antigen on B-lymphocytes, selectively depleting these cells. Furthermore, rituximab seems to have a direct protective effect on podocytes. Rituximab partially prevented the downregulation of sphingomyelin phosphodiesterase acid-like 3b (SMPDL-3b) protein and acid sphingomyelinase (ASMase) that was observed in podocytes treated with the sera of patients with recurrent FSGS [55]. Its beneficial effect on recurrent posttransplant FSGS was reported initially in 2006 [56]. Since then several reports on the efficacy of rituximab treating recurrent FSGS have been published (summarized in [39]). A systematic review of 39 reported cases indicates a complete or partial remission in 64% of the patients [57]. Normal serum albumin at recurrence and younger age were associated with treatment response. Interestingly, in the univariate analysis fewer rituximab infusions were also associated with a better treatment effect, which is in line with data from idiopathic membranous nephropathy and its recurrence after transplantation [58]. It remains to be elucidated if titrating the dose of rituximab to obtain B-cell depletion is the optimal strategy in this setting. In the published reports a typical rituximab regimen is 2–6 doses of 375 mg/m^2^/dose given once every one to two weeks. Some case reports suggested better efficacy when rituximab is combined with plasmapheresis [59, 60]. In the reports of 4 children by Tsagalis et al., rituximab was administered at a dose of 1 g, two doses two weeks apart. After rituximab infusion, plasmapheresis was not performed during the next 72 hours to prevent removal of the antibody. Complete remission was achieved in 2 and partial remission in the other 2 patients, while renal function remained stable and no severe infectious complications occurred during a follow-up time of 18–60 months [59].

Despite evidence that activation of the renin-angiotensin system (RAS) is also crucially involved in progression of recurrent FSGS [61], only few case reports have been published addressing the beneficial effect of RAS blockade on proteinuria reduction in recurrent FSGS [50, 62, 63]. Reduction of proteinuria in this setting nicely illustrates that recurrent FSGS is not entirely immunologically mediated but rather includes components of both primary and adaptive FSGS.

In one case report an intravenous infusion of galactose in a patient with recurrent FSGS reduced circulating permeability factor activity [64]. The current use of cyclophosphamide in recurrent FSGS is not frequent due to contradictory results and concerns about long-term toxicity [25]. In a case series of 4 patients with recurrent FSGS resistant to plasmapheresis and rituximab treatment with abatacept, a soluble fusion protein that blocks the T-cell costimulatory protein B7-1 (CD80) was associated with almost complete remission [65]. However, others were not able to reproduce these beneficial results neither with abatacept nor with belatacept [66–68].

## 5. Living Kidney Donation in Patients with FSGS

Data from large registries such as the Recurrent Allograft Disease Registry (RADR), the USRDS, the North American Pediatric Renal Transplant Cooperative Study (NAPRTCS), and the UNOS demonstrated a similar rate of FSGS recurrence and graft loss among recipients of kidneys from living versus deceased donors [7, 10, 69, 70]. However, recipients of living donor transplants may lose their survival advantage which is usually observed over recipients of deceased donor transplants [69]. Analysis of the USRDS data showed that death-censored graft survival was significantly better in patients with FSGS who received a zero-mismatch kidney from living donors as compared to patients who received a zero-mismatch organ from cadaveric donors [71]. In the case of genetic or familial FSGS, donor selection has to be performed with great caution. In the cohort examined by Jungraithmayr et al., not only homozygous NPHS2 mutations but also heterozygous NPHS2 mutations or variants (e.g., R229Q) were identified. The authors proposed to perform genetic screening of the related donor when the recipient has a mutation of NPHS2. In the case of recessive (i.e., homozygous) mutations, one could accept a heterozygous donor. In the case of heterozygous mutations or variants such as R229Q, it is not clear if this is a dominant-negative variant, which would pose the donor at risk. In such case the donor should not be accepted. In particular the long-term clinical significance of heterozygous NPHS2 mutations is currently unknown, and some centers do not accept heterozygote donors.

## 6. Conclusions

The frequent recurrence of FSGS in the allograft is associated with poor graft survival. Despite novel insights into the pathogenesis of FSGS and its recurrence, outcomes did not substantially change in the last decade. Plasmapheresis, high-dose cyclosporine, and more recently rituximab represent the most promising therapeutical options. Recent advances on the pathology and pathophysiology of podocyte injury in FSGS, some of them hypothesis-driven and some hypothesis-generating, may transform not only into better risk-stratification, but also into more specific therapies for patients with FSGS.
